# Treatment of Monosymptomatic Nocturnal Enuresis: Sertraline for Non-Responders to Desmopressin

**Published:** 2014-03

**Authors:** Reza Mahdavi-Zafarghandi, Ali Seyedi

**Affiliations:** Department of Urology, Imam Reza Educational Hospital, Faculty of Medicine, Mashhad University of Medical Sciences, Mashhad, Iran

**Keywords:** Nocturnal enuresis, Sertraline, Adolescent

## Abstract

One of the challenges in the management of primary monosymptomatic enuresis (PME), especially in adolescents, is response failure to medical regimens such as desmopressin. This prospective study aimed at addressing the efficacy of sertraline in the treatment of 25 adolescents (13-18 year old) with PME having experienced failure to previous desmopressin therapy. Patients were recommended to take one oral tablet of sertraline (50 mg) every morning after meal for 3 months. The patients were followedup every 6 weeks, the final visit being 6 months after treatment termination. Comparing the number of wet nights in the pretreatment nocturnal records with the follow-up visits revealed a significant reduction (P=0.01). The primary efficacy outcome was achieved in 18 (72%) of the 25 patients; 12 patients had full response, whereas six patients showed partial response. Four (16%) of the 25 children presented with a relapse after 6 months of follow-up. Drug-related adverse events were rare. Sertraline effectively reduced the number of wet episodes in adolescents with PME who had experienced failure to desmopressin therapy. With respect to the favorable efficacy outcome of this medication and the scarce drug-related adverse effects, sertraline can be proposed as a novel treatment for PME.

## Introduction

Nocturnal enuresis refers to involuntary voiding only at night, above the age at which most children have stopped.^1^ At least 3 occasions of bedwetting in a patient who has never been dry for longer than 6 months is approved for the diagnosis of primary monosymptomatic enuresis (PME).^2^^,^^3^ Despite the maturation rate of 15% per year, 0.5% of all cases persist in adulthood, with notable consequences on self-esteem.^4^^,^^5^ Numerous treatment regimes for PME have been proposed, including behavioral and motivational therapy, alarm aid, and pharmacotherapy.^6^ Medical treatment of PME mainly consists of either desmopressin or antimuscarinics such as propiverine or oxybutynin.^7^^,^^8^ One of the challenges in the management of PME is response failure to these pharmaceuticals.

The effect of drugs which manipulate serotonin levels such as selective serotonin reuptake inhibitors (SSRIs) on urination has been noted in recent literature.^9^ These data suggest that SSRIs may become new drugs for the treatment of nocturnal enuresis without the serious cardiac arrhythmia associated with tricyclic antidepressants or the hyponatremia associated with long-term desmopressin treatment. Our study aimed at evaluating the efficacy of sertraline in the treatment of adolescent patients with enuresis who had failed to respond to former desmopressin therapy.

## Patients and Methods

From March 2009 to April 2011, adolescents with PME refractory to desmopressin at the maximal dosage of 0.6 mg per night who were referred to Imam Reza Educational Hospital, Mashhad, Iran were enrolled consequentially in this prospective before-after study. Failure to desmopressin was described as a 0% to 49% decrease in the number of wet nights per week.^10^ The sample size was estimated on the basis of the number of wet nights for patients undergoing treatment with sertraline. For sample size calculation, mean±standard deviation was used based on Sukhai et al’s.^11^ study. Considering α=0.05 and β=0.2, the sample size was calculated as 25. All the cases had more than 4 wet nights per week. A full medical history was obtained and a targeted physical examination was performed, comprising neuro-urological condition and a 3-day, 24-hour, frequency-volume chart to rule out lower urinary tract dysfunction. Patients were excluded from the study if any urinary symptoms or bowel elimination difficulties (e.g., encopresis constipation) were noted in their medical history. Urinary tract infections and other organic causes were excluded by urine culture and analysis and ultrasonographic examination of the kidneys and bladder. A one-week nocturnal record was also collected from each patient to determine the number of wet nights. All of these data were collected over a 2 to 3-week period before study entrance. An informed consent was signed by each patient, and the study protocol was approved by the local Ethics Committee of Mashhad University of Medical Sciences (#2312566).

The patients were recommended to take one oral tablet of sertraline (50 mg) every morning after a meal for 12 weeks. At the end of the third month, the drug was tapered by 25 mg every 2 weeks (4 weeks in total). Follow-up visits were done every 6 weeks during the 3-month treatment period, evaluating efficacy, adverse events and relapse of symptoms. The final follow-up visit was 6 months after treatment termination (9 months after study initiation). The patients were instructed to report the number of wet nights and doses of medications given. Comparison between the number of wet nights in the pretreatment nocturnal records and the number of those during the follow-up visits was used to demonstrate the efficacy of the therapy. The treatment results were categorized as “success” or “no success” on the basis of the one-week nocturnal records collected at the end of the treatment period. Successful outcomes included the following responses, as defined by the Standardization Committee of the International Children’s Continence Society:^10^ full response, no wet nights; response, 90% reduction in the number of wet nights; and partial response, 50% to 89% reduction in the number of wet nights. An unsuccessful outcome was defined as no response (50% reduction in the number of wet nights). Relapse, denoted as more than one wet night/month at 6 months after sertraline termination, was the secondary efficacy outcome.

The collected data were then analyzed using the Statistical Package of Social Science (SPSS Inc., Chicago, IL) for Windows (version 11.5). A P<0.05 was considered statistically significant. The one-sample Kolmogorov-Smirnov test was used for the quantitative analysis of the normalcy of the variables. Comparative analysis was subsequently carried out at 6 weeks, and after 3, 6, and 9 months, using repeated measures. 

## Results

**Figure 1:**
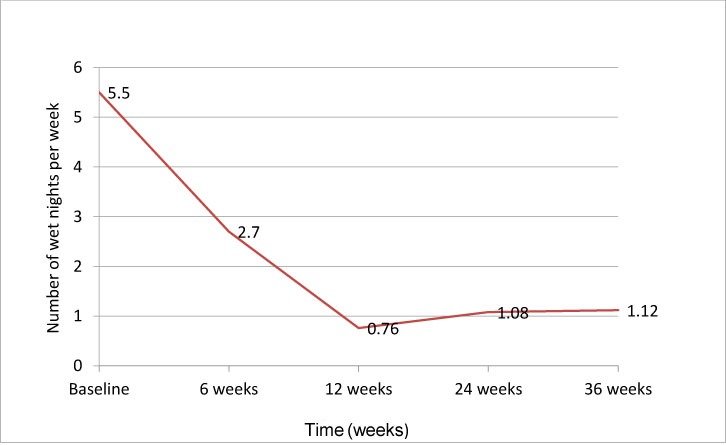
Mean number of wet nights after treatment with sertraline.

This study was designed to examine patients with PME refractory to desmopressin. 25 patients aged 13-18 years (mean±SD=15.48±1.5 yrs; 11 girls, 14 boys) met the inclusion criteria. After 6 weeks of therapy, a significant reduction in the mean number of wet nights was found (figure 1). The primary efficacy outcome was achieved in 18 of the 25 (72%) patients 9 months after sertraline initiation. Six patients had a partial response, defined as ≥50% but ≤90% reduction in wet nights. Overall, 4 of the 25 patients (16%) presented with a relapse after 6 months of follow-up. No drug-emergent adverse events were observed.

Only 3 of the 25 patients had adverse reactions of moderate intensity, requiring no early treatment cessation. The affected patients reported headache and nausea. 

By using a generalized estimating equation approach, the risk of wet episodes per night was compared showing a significant decrease of 74% in the risk of a wet episode in the study group.

## Discussion

MacLean^12^ noticed that imipramine, a tricyclic antidepressant, improved patients’ enuresis. Mesaros^13^ discovered the therapeutic effect of SSRIs on nocturnal enuresis, when treating dysthymia. Kano proposed Fluvoxamine as a possible drug for the treatment of enuresis with respect to his experience in patients with obsessive-compulsive disorder.^9^

Toren and collegues^14^ were the first to evaluate the efficacy of Fluvoxamine in the treatment of enuresis in children and adolescents. In their case series, no improvement in the mean voiding frequency of patients was observed. Conversely, 4 of 9 patients showed a trend toward an increase in the frequency of enuresis during treatment. The author concluded that fluvoxamine had no anti-diuretic properties. However, the small number of subjects and mixed target population of the patients should be considered.

In the current study, the effect of sertraline was investigated in adolescent PME patients who had failed to respond to previous desmopressin therapy. The frequency of enuresis decreased in 18 (72%) patients. 

Water intoxication is a rare but serious side effect associated with desmopressin.^15^ Imipramine has important adverse effects, and overdose can be lethal. The known side effects of sertraline include sleep disturbance, headache, tremors, agitation, and gastrointestinal upset. In the current investigation, drug adverse effects were observed only in 3 patients, which did not warrant withdrawal from the study.

This study demonstrates that sertraline could be of value in some PME in whom previous conventional therapy with desmopressin has failed. To our knowledge, this treatment modality has not been tried before in such cases. However, at 6-month follow-up off-sertraline, we detected some deterioration in the response rate compared to early results and 4 (16%) patients experienced relapse. This deterioration during follow-up suggests that sertraline may have temporary efficacy and its effect may decrease gradually with time.

It has been noted in the medical literature that serotonin level alteration has specific effects on urination. Serotonin inhibits ureteral peristalsis as well as micturition by interfering with spinal reflexes, primarily through 5-HT3 receptor agonism. The central effects of serotonin on urination are more complicated. For instance, while the antagonism of presynaptic 5-HT1A receptors has an inhibitory effect on bladder reactivity, their agonists appear to diminish the threshold for micturition.^16^^,^^17^ However, it should be remembered that micturition and enuresis are multifactorial processes and a number of various neurotransmitters and neuropeptides beyond serotonin are involved.

The main limitations of this study are the small sample size and the short 6-month follow-up period. In addition, we did not perform urodynamic tests before the initiation of sertraline or at more follow-up visits to avoid patient discomfort. However, this information could be important for assessing the cause of deterioration in sertraline efficacy after the treatment was stopped. 

Sertraline appears to be a viable treatment option in patients with refractory PME given its fewer adverse effects and higher validity in comparison to Imipramine. This report supports the previous evidence suggesting a serotonergic mechanism in enuresis, which may be at least partially independent of the serotonergic mechanism of mood disorders. Nevertheless, deterioration in some responders with time raises important questions about the long-term efficacy of this therapy and the need for further maintenance sessions. More studies are needed to support our findings and select patients who would be suitable candidates for this therapy.

## Conclusion

Sertraline was well tolerated by the study participants, and our results support the use of SSRIs in the treatment of enuresis. The findings of this pilot study provide feasible data to recommend larger randomized controlled trials in order to examine the efficacy of sertraline in the management of enuresis.

## References

[1] Eberdt-Gołąbek  B, Zmysłowska K, Słowik M, Hozyasz K (2013). Etiology primary, monosymptomatic nocturnal enuresis in children. Own research. Med Wieku Rozwoj.

[2] Kiddoo DA (2012). Nocturnal enuresis. CMAJ.

[3] Nevéus T (PubMed PMID: 21267599). Nocturnal enuresis-theoretic background and practical guidelines. 2011;26:1207-14.

[4] Landgraf JM, Abidari J, Cilento BG, Cooper CS, Schulman SL, Ortenberg J (2004). Coping, commitment, and attitude: quantifying the everyday burden of enuresis on children and their families. Pediatrics.

[5] Vande Walle, Rittig S, Bauer S, Eggert P, Marschall-Kehrel D, Tekgul S (2012). Practical consensus guidelines for the management of enuresis. Eur J Pediatr.

[6] Austin PF, Ferguson G, Yan Y, Campigotto MJ, Royer ME, Coplen DE (2008). Combination therapy with desmopressin and an anticholinergic medication for nonresponders to desmopressin for monosymptomatic nocturnal enuresis: a randomized, double-blind, placebo-controlled trial. Pediatrics.

[7] Lee T, Suh HJ, Lee HJ, Lee JE (2005). Comparison of effects of treatment of primary nocturnal enuresis with oxybutynin plus desmopressin, desmopressin alone or imipramine alone: a randomized controlled clinical trial. J Urol.

[8] Marschall-Kehrel AD, Mürtz G, Kramer G, Jünemann KP, Madersbacher H, Hjalmas K (2004). A suggested treatment algorithm in nocturnal enuresis with emphasis on partial responders. Urologe A.

[9] Kano K, Arisaka O (2003). Relationship between fluvoxamine and stress barometer for nocturnal enuresis. Pediatr Int.

[10] Nevéus T, von Gontard, Hoebeke P, Hjälmås K, Bauer S, Bower W (2006). The standardization of terminology of lower urinary tract function in children and adolescents: report from the Standardisation Committee of the International Children's Continence Society. J Urol.

[11] Sukhai RN, Mol J, Harris AS (1989). Combined therapy of enuresis alarm and desmopressin in the treatment of nocturnal enuresis. Eur J Pediatr.

[12] MacLean RE (1960). Imipramine hydrochloride (Tofranil) and enuresis. Am J Psychiatry.

[13] Mesaros JD (1993). Fluoxetine for primary enuresis. J Am Acad Child Adolesc Psychiatry.

[14] Toren P, Eldar S, Laor N, Wolmer L, Samuel E, Weizman R (2001). FFluvoxamine is ineffective in the treatment of enuresis in children and adolescents: an open-label pilot study. Hum Psychopharmacol.

[15] Larney V, Dwyer R (2006). Hyponatraemic convulsions and fatal head injury secondary to desmopressin treatment for enuresis. Eur J Anaesthesiol.

[16] Cheng CL, de Groat (2010). Role of 5-HT1A receptors in control of lower urinary tract function in anesthetized rats. Am J Physiol Renal Physiol.

[17] Mbaki Y, Ramage AG (2008). Investigation of the role of 5-HT2 receptor subtypes in the control of the bladder and the urethra in the anaesthetized female rat. Br J Pharmacol.

